# Novel missense mutations in exon 15 of desmoglein-2: Role of the intracellular cadherin segment in arrhythmogenic right ventricular cardiomyopathy?

**DOI:** 10.1016/j.hrthm.2010.08.007

**Published:** 2010-10

**Authors:** Katja Gehmlich, Angeliki Asimaki, Thomas J. Cahill, Elisabeth Ehler, Petros Syrris, Elisabetta Zachara, Federica Re, Andrea Avella, Lorenzo Monserrat, Jeffrey E. Saffitz, William J. McKenna

**Affiliations:** ⁎ Institute of Cardiovascular Science, University College London, United Kingdom; † Department of Pathology, Beth Israel Deaconess Medical Center, Harvard Medical School, Boston, Massachusetts, United States; ‡ King's College London, The Randall Division of Cell and Molecular Biophysics and the Cardiovascular Division, BHF Centre of Research Excellence, London, United Kingdom; § Cardiovascular Department, Cardiomyopathies Unit, St. Camillo Hospital, Rome, Italy; ∥ Cardiovascular Department, Arrhythmia Unit, Camillo Hospital, Rome, Italy; ¶ Cardiology Department, Cardiomyopathies Unit, Complejo Hospitalario Universitario de A Coruña, A Coruña, Spain

**Keywords:** Arrhythmogenic right ventricular cardiomyopathy, Desmoglein-2, Desmosome, Genetics, Missense mutation, Plakophilin-2, ARVC, arrhythmogenic right ventricular cardiomyopathy, Cx43, connexin43, DSC2, desmocollin-2, DSG2, desmoglein-2, DSP, desmoplakin, GFP, green fluorescent protein, GST, glutathione-S-transferase, ICS, intracellular cadherin segment, PG, plakoglobin, PKP2, plakophilin-2, RV, right ventricle

## Abstract

**Background:**

The diagnosis of arrhythmogenic right ventricular cardiomyopathy can be challenging. Disease-causing mutations in desmosomal genes have been identified. A novel diagnostic feature, loss of immunoreactivity for plakoglobin from the intercalated disks, recently was proposed.

**Objective:**

The purpose of this study was to identify two novel mutations in the intracellular cadherin segment of desmoglein-2 (G812S and C813R in exon 15). Co-segregation of the G812S mutation with disease expression was established in a large Caucasian family. Endomyocardial biopsies of two individuals showed reduced plakoglobin signal at the intercalated disk.

**Methods:**

To understand the pathologic changes occurring in the diseased myocardium, functional studies on three mutations in exon 15 of desmoglein-2 (G812C, G812S, C813R) were performed.

**Results:**

Localization studies failed to detect any differences in targeting or stability of the mutant proteins, suggesting that they act via a dominant negative mechanism. Binding assays were performed to probe for altered binding affinities toward other desmosomal proteins, such as plakoglobin and plakophilin-2. Although no differences were observed for the mutated proteins in comparison to wild-type desmoglein-2, binding to plakophilin-2 depended on the expression system (i.e., bacterial vs mammalian protein expression). In addition, abnormal migration of the C813R mutant protein was observed in gel electrophoresis.

**Conclusion:**

Loss of plakoglobin immunoreactivity from the intercalated disks appears to be the endpoint of complex pathologic changes, and our functional data suggest that yet unknown posttranslational modifications of desmoglein-2 might be involved.

## Introduction

Arrhythmogenic right ventricular cardiomyopathy (ARVC) is a disease of the heart muscle. It is characterized by life-threatening arrhythmias and heart failure of predominantly right ventricular (RV) origin.^1^ Several factors, including marked phenotypic variation, incomplete and low (30%) penetrance, and age-related disease development and progression contribute to the complexity of clinical diagnosis.^2^ Familial disease occurs in approximately 50% of cases, and five genes coding for cardiac desmosomal proteins are associated with the disease.^3^

It is widely accepted that changes in cellular adhesion underlie ARVC pathogenesis.^3^ The redistribution of plakoglobin (PG) from junctional to intracellular pools (mirrored by significant reduction in the amount of immunoreactive signal at cardiac intercalated disks) has been reported in virtually all patients with ARVC irrespective of the underlying pathogenic mutation.^4^ The mechanisms responsible for this consistent immunohistochemical feature are currently being investigated. Preliminary data have shown that PG redistribution may be related to signal transduction pathways that appear to be pathologically activated.^5^

Impaired desmosomal structure and function also affect other cell-to-cell contact structures in the myocardium. In particular, gap junction remodeling is often observed in ARVC patients, and these potential electrical conduction abnormalities may favor arrhythmic events that characterize the disease and predispose patients to high risk of sudden cardiac death.^6,7^

Desmoglein-2 (DSG2) and desmocollin-2 (DSC2) are the cardiac desmosomal cadherins, which mediate adhesion across cardiomyocytes via their extracellular domains.^8^ Their cytoplasmic portions have a conserved domain, the intracellular cadherin segment (ICS). This 60-amino-acid-long module provides the link to the intracellular desmosomal components PG and plakophilin-2 (PKP2), which in turn bind desmoplakin (DSP) and thereby provide a link to the desmin filaments.^9^

Approximately 10% of patients with ARVC have mutations in DSG2. In the intracellular cytoplasmic portion only two mutations (DSG2 G812C and DSG2 V920G) have been described; however, the latter is also found in 0.8% of the healthy population, making a causative role in ARVC doubtful.^10^

Here we present two novel missense mutations in the DSG2 gene, residing in the immediate region of the previously identified G812C mutation.^11^ Co-segregation with the disease phenotype could be established in the case of one mutation. Immunohistochemical analysis and functional *in vitro* studies are supportive of a causal role for the mutated DSG2 ICS domain in ARVC. Furthermore our data point toward complex mechanisms underlying disease pathogenesis.

## Methods

### Clinical evaluation and genetic screening

The study was performed in accordance with the 1964 Declaration of Helsinki, and the study protocol was approved by the local ethics committees. All subjects provided informed consent. Clinical evaluation and genetic screening of DSP, PG, PKP2, DSG2, DSC2, and connexin43 (Cx43) by direct sequencing were performed as described previously.^12^ The diagnosis of ARVC was based on the recent revision of the original criteria proposed by the International Task Force of the European Society of Cardiology and International Society and Federation of Cardiology.^13^

### Analysis of endomyocardial biopsies

RV biopsy samples obtained from the proband (A.1) and his sister (A.3) during a right heart catheterization were histologically analyzed or immunostained as previously described.^14^

### Functional studies

For localization and expression studies, full-length human DSG2 constructs fused to green fluorescent protein (GFP) were expressed in neonatal rat cardiomyocytes using pEGFP-N1 vector (Clontech, Takara Bio Europe, Saint-Germain-en-Laye, France). For binding assays, DSG2 ICS sequences (amino acids 635–842 of human DSG2) were used in glutathione-*S*-transferase (GST) pulldown assays. Full experimental details are available in the online supplementary material.

## Results

### Identification of novel DSG2 missense mutations in families with ARVC

**Figure 1:**
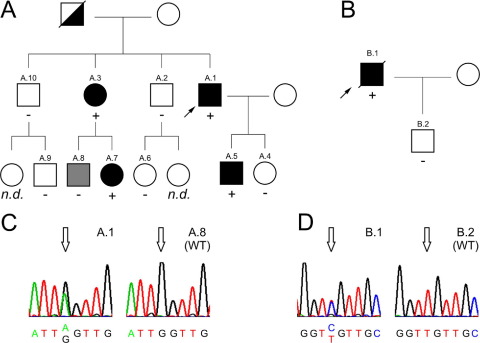
Identification of novel mutations in DSG2 exon 15. **A:** Pedigree of family A with a heterozygous DSG2 G812S mutation. *Black symbols* indicate individuals fulfilling diagnostic criteria for arrhythmogenic right ventricular cardiomyopathy (ARVC)^1^; *white symbols* indicate individuals who do not fulfill task force criteria; *gray symbol* indicates borderline ARVC diagnosis; *black and white symbol* indicates sudden cardiac death at age 42 years. *Slanted bars* indicate deceased individuals; *squares* indicate males; *circles* indicate females; *plus* and *minus signs* indicate presence or absence of DSG2 mutation, respectively; *arrow* indicates index patient. n.d. = individual not available for clinical evaluation. For details of the clinical evaluation, see Table 1. **B:** Pedigree of family B with a heterozygous DSG2 C813R mutation. Symbols as in panel A. **C:** Sequence electropherogram of the G→A change at nucleotide position c. 2434 (*open arrow* in individual A.1) compared to a normal wild-type (WT) individual (A.8). **D:** Sequence electropherogram of the T→C change at nucleotide position c. 2437 (*open arrow* in individual B.1) compared to a normal WT individual (B.2).

The index patient of a Caucasian family (individual A.1 of family I, Figure 1A) presented with palpitations at age 37 years. Clinical evaluation identified 35,000 ventricular ectopic beats on 24-hour ECG monitoring and structural abnormalities of the RV on echocardiogram. Endomyocardial biopsy was performed targeting RV low-voltage areas identified by electroanatomic mapping.^15^ Histologic analysis of the myocardial sample showed clear evidence of interstitial and replacement fibrosis (Online Supplement Figure 1). The diagnosis of ARVC was made on the basis of two major and one minor criteria according to the revised task force criteria (Table 1).^13^

Genetic screening of the five cardiac desmosomal genes and Cx43 showed no abnormalities in DSP, PKP2, DSC2, PG, or Cx43. In DSG2, a heterozygous G→A change was found in exon 15 at position c.2434, coding for a glycine to serine amino acid change at position 812 (DSG2 G812S; Figure 1C).

The father of the index patient had died suddenly at age 42 years and had experienced palpitations and presyncope in the past. Clinical evaluation of the family showed that the index patient's sister (individual A.3) also fulfilled diagnostic criteria for ARVC (three major and one minor criteria; Table 1) in the presence of the DSG2 G812S mutation (severe dilation and reduction of RV ejection fraction on echocardiogram, with RV outflow tract parasternal short-axis measurement of 38 mm; 700 ventricular ectopic beats on 24-hour ECG monitoring; fibrofatty replacement on endomyocardial biopsy). The brothers of the index patient (individuals A.2 and A.10) had normal ECGs and normal echocardiography findings. They were negative for the mutation, and the same applied to all of their children available for clinical testing (individuals A.6 and A.9). The children of both affected individuals were also investigated. Two of them (individuals A.5 and A.7) were found to carry the mutation and were diagnosed with ARVC (based on two major criteria, akinesia of the RV apex with RV outflow tract parasternal short-axis measurement of 36 mm; Table 1). The daughter of the index patient (individual A.4) was negative for the mutation and was clinically normal according to ECG and echocardiographic investigation. Individual A.8 was negative for the mutation; however, his echocardiogram showed RV apical hypokinesia; therefore, he is also classified as borderline for ARVC (one major and one minor criteria; Table 1).

In a second Caucasian family, the index patient (individual B.1, Figure 1B) presented with symptomatic sustained ventricular tachycardia (presyncope and sweating) at age 76 years and had previously complained of palpitations. Clinical sustained monomorphic ventricular tachycardia with left bundle branch block morphology and superior axis was documented. His resting ECG showed T-wave inversion in leads V_1_–V_3_ and right bundle branch block. Echocardiography demonstrated RV dilation with small aneurysmal areas of the free wall and moderate RV systolic dysfunction, a nondilated left ventricle with normal systolic function, mild concentric hypertrophy, and mild left atrial dilation. The diagnosis of ARVC was based on two major and one minor criteria (Table 1). The patient died of cancer at age 77 years.

Genetic screening identified a heterozygous base pair change in DSG2 (c.2437 T→C change in exon 15), coding for a cysteine to arginine substitution at amino acid 813 (DSG2 C813R missense mutation; Figure 1D). No changes were found in the other four cardiac desmosomal genes or Cx43.

There was no previous family history of cardiomyopathy or sudden death. The only son of the index patient (individual B.2) was negative for the mutation and was clinically normal. No siblings of the index patient were available for clinical or genetic investigation.

### Immunohistochemical changes in the presence of a DSG2 missense mutation

**Figure 2:**
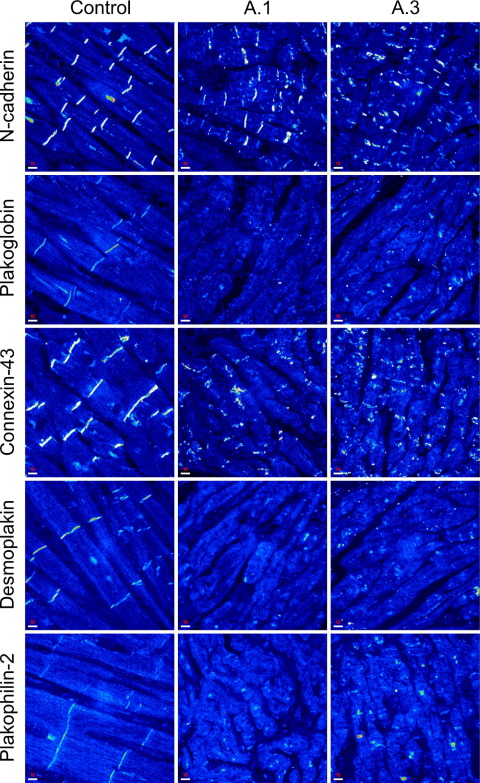
Confocal immunofluorescence microscopy analysis of control and gene-positive right ventricular myocardium (individuals A.1 and A.3; see Figure 1) showing the amount of immunoreactive signal for selected junctional proteins at intercalated disks. Scale bars (bottom left) = 10 μm.

Endomyocardial biopsy material was available from two gene-positive individuals of family A (Figure 2). Immunohistochemical analysis of both samples showed significantly reduced expression of PG at cardiac intercalated disks. Moreover, immunoreactive signal for two other desmosomal proteins (DSP and PKP2) was also significantly depressed, whereas signal for the nondesmosomal adhesion molecule N-cadherin was present and indistinguishable from controls. Furthermore, expression of the major ventricular gap junction protein Cx43 was severely reduced at cardiac intercalated disks of both affected individuals. Based on the recently presented novel diagnostic approach, normal immunoreactive signal for N-cadherin and reduced signal for PG were consistent with previously described molecular changes occurring in ARVC.^4^

### Novel DSG2 missense mutations affect a conserved region

Both novel missense mutations DSG2 G812S and DSG2 C813R were absent in 400 control individuals. Further supporting evidence for a pathogenic role comes from a report of a similar mutation at amino acid position 812 of DSG2 (heterozygous glycine to cysteine change: DSG2 G812C), which has been found to be causative for ARVC in a U.S. patient.^11^

**Figure 3:**
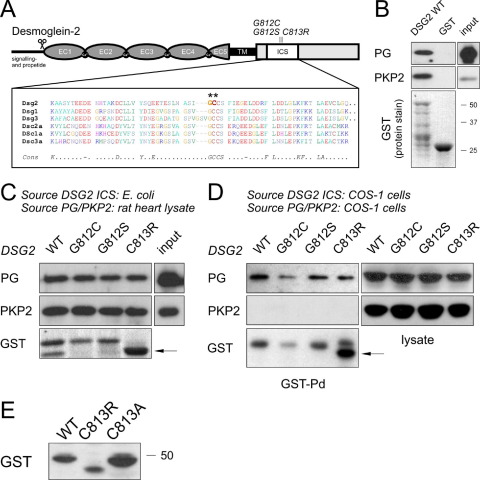
Mutations in the desmoglein-2 (DSG2) intracellular cadherin segment (ICS) domain. **A:** Position of the mutations in DSG2. (Modified from Awad et al.^3^) The proteolytic cleavage site *(scissors symbol)*, the five extracellular cadherin domains (EC), the transmembrane region (TM), and the location of the ICS domain within the cytoplasmic portion are indicated. Sequence alignment of the ICS domain for desmosomal cadherin demonstrates conservation of the affected amino acids G812 and C813 *(asterisks).***B:** Binding of plakoglobin (PG) and plakophilin-2 (PKP2) to DSG2 ICS wild-type (WT) in glutathione-*S*-transferase (GST) pulldown assays. A 1% input dilution of the rat heart lysate and GST-fusion protein input, together with GST alone control, are shown (position of marker proteins is indicated on the *right*). Multiple bands below 50 kD in lane 1 are due to partial degradation of DSG2 ICS *in vitro.***C:** In binding experiments similar to those shown in panel B, the binding of the three DSG2 mutations was tested in comparison to DSG2 ICS WT. No changes were observed in binding of PG and PKP2. The abnormal migration of DSG2 C813R is marked with an *arrow.***D:** Binding experiments with GST fusion proteins expressed in mammalian COS-1 cells. Binding to PG was normal for all three mutants tested, whereas binding to PKP2 was below the detection limit. *Arrow* as in panel C. **E:** Altered electrophoretic mobility of DSG2 ICS C813R in comparison to the WT protein fragment. A DSG2 ICS C813A mutation restores normal migration properties.

The accumulation of three ARVC mutations at two adjacent amino acids suggested that this region of DSG2 might be crucial for desmosomal function. Both residues glycine 812 and cysteine 813 are part of the completely conserved core module in the ICS domain (Figure 3A), which is the domain that mediates binding of the desmosomal cadherins to PG.^16^ This implies that the three mutations in this region may interfere with protein functions (e.g., binding to PG) and may act via the same disease pathway.

Our binding experiments confirmed that the ICS of DSG2 links the molecule to other desmosomal components. A bacterially expressed, purified GST fusion protein fragment corresponding to the DSG2 ICS bound to both PG and PKP2 from rat heart lysates (Figure 3B), whereas GST alone showed no binding. Compared to the signal intensity of the input control, binding to PKP2 was stronger than binding to PG.

### Properties of cytoplasmic DSG2 missense mutations

**Figure 4:**
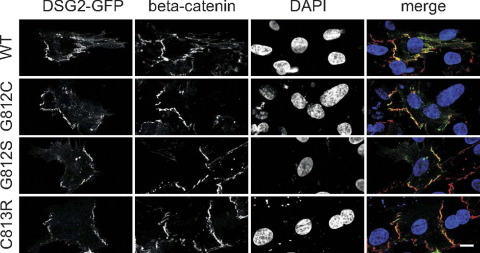
Localization of the three desmoglein-2 (DSG2) mutant proteins at the intercalated disk of cardiomyocytes. Green fluorescent protein (GFP) fusion proteins of DSG2 wild-type (WT) and the three mutants were transiently expressed in NRC. Intercalated disks were visualized by counterstaining for beta-catenin. Nuclei were visualized with 4′,6-diamidino-2-phenylindole (DAPI). Merged images: DSG2-GFP *green*, beta-catenin *red*, DAPI *blue.* Scale bar = 10 μm.

In transient transfection experiments using GFP fusion proteins, all three mutant DSG2 proteins were normally incorporated into the intercalated disk structures of primary neonatal rat cardiomyocytes (Figure 4). Furthermore, none of the three mutations affected protein stability or turnover (data not shown).

To study potential consequences of the mutations on binding properties, the three mutations were introduced into the GST-DSG2 ICS fusion construct and used in GST pulldown assays. No binding differences were observed, and all three mutant proteins bound PG or PKP2 with an affinity comparable to DSG2 wild-type (Figure 3C).

We speculated that posttranslational modifications of the DSG2 ICS segment may occur *in vivo*, which could modulate binding to other desmosomal proteins, and that the three mutations might influence such posttranslational modifications. Therefore, the same DSG2 ICS protein fragments were expressed as GST fusion proteins in mammalian COS-1 cells and probed for binding to endogenous PG and PKP2 in GST pulldown assays. All three mutants bound to PG like the wild-type, and no significant differences in the binding affinity were observed (Figure 3D). However, and in contrast to binding experiments with bacterially expressed protein fragments, no binding to PKP2 was observed, not even for the wild-type protein (Figure 3D).

The DSG2 C813R protein fragment migrated reproducibly faster on polyacrylamide gels than did its wild-type counterpart (arrows in Figures 3C and 3D). To test whether this was due to the newly introduced arginine residue in the mutant (“gain of function,” e.g., due to the additional positive charge) or the lack of the cysteine side chain, a second mutant protein was generated that contained the nonpolar alanine residue instead of the cysteine or arginine side chain at the same position (DSG2 C813A). The introduction of this alanine mutation restored normal migration properties on polyacrylamide gels (Figure 3E), suggesting that the arginine side chain in the mutant is responsible for the observed electrophoretic abnormalities.

These data suggest that posttranslational modifications of the DSG2 ICS domain may occur in mammalian cells, which might modulate DSG2 functions.

## Discussion

ARVC is a cardiac muscle disorder that is associated with arrhythmias and heart failure of predominantly RV origin. Molecular genetics postulate ARVC to be a “disease of the desmosome.”^17^ However, the underlying molecular pathogenesis and clinical characteristics, such as variable disease expression and incomplete penetrance, are poorly understood.

Here we report two novel missense mutations in the ICS, a conserved region in the cytoplasmic domain of DSG2 (DSG2 G812S and DSG2 C813R) identified in two ARVC probands. The first mutation affects the same residue as the previously reported DSG2 G812C mutation, which was found in an American family,^11^ while the second is in the subsequent amino acid. Co-segregation of the missense mutation DSG2 G812S with disease expression supports its causative role for ARVC in family A (Figure 1). The second family with the DSG2 C813R mutation was too small for co-segregation analysis.

Other mutations in DSG2 have been associated with ARVC.^11,18-20^ Most of the mutations are located in the extracellular portion of the protein, but no clear correlation has been observed between specific mutations and clinical features. Due to the small number of affected individuals in our study and the observed variable disease expression, we currently cannot draw any conclusions regarding differences in phenotypes of individuals bearing extracellular or cytoplasmic DSG2 mutations.

A few functional studies on the molecular pathology of DSG2 mutations in ARVC have been reported. A DSG2 N266S transgenic mouse model, which mimics the human mutation DSG2 N271S in the extracellular domain of the protein, has been studied.^20^ This animal showed some typical features of ARVC, such as ventricular arrhythmias and sudden cardiac death. At the histologic level, necrosis was observed in the transgenic hearts; however, desmosomal structure in electron micrographs and immunohistochemical staining for PG, PKP2, DSP, and Cx43 at the intercalated disk appeared to be normal in this animal model.

This finding contrasts with the molecular phenotype observed in our patients with a novel mutation in the C-terminus of DSG2. The reduction of immunoreactive signal specific for the desmosomal proteins DSP, PG, and PKP2 from cardiac intercalated disks in two individuals with the DSG2 G812S mutation (Figure 2), but not in the mouse model, may potentially be explained by differences between murine ARVC models and human disease. Similarly, the heterozygous PG knockout mice reflected only certain aspects of human disease (arrhythmias and reduced RV function), whereas no fatty fibrotic replacement of cardiomyocytes was evident in this animal model for ARVC.^21^

The electric isolation of cardiomyocytes by surrounding scar tissue documented in individual A.1 may promote reentrant excitation (Online Supplement Figure 1). Moreover, the gap junction remodeling observed in the endomyocardial biopsy samples (indicated by reduced immunoreactive signal for Cx43, Figure 2) may act synergistically with the histologic abnormalities characteristic of ARVC to enhance conduction heterogeneity and increase the risk of arrhythmia.^7^ Because loss of PKP2 was evident in the samples (Figure 2), PKP2-associated conduction slowing (via Cx43^22^ or sodium channels^23^) may also contribute to the development of arrhythmia.

Our functional studies on three ARVC-associated mutations in the cytoplasmic domain of DSG2 suggest that these mutant proteins act in a dominant negative manner. The localization and stability of the mutant proteins were identical to DSG2 wild-type (Figure 4), suggesting that the mutant proteins are likely to be incorporated into the cardiac desmosomes of the patients. The fact that all mutations cluster in the ICS implies an important role of this domain for DSG2 function. The affected residues are completely conserved among desmosomal cadherins (Figure 3A), and the ICS domain links DSG2 to the plaque proteins PKP2 and PG^9^ (Figure 3B). Contrary to our hypothesis (see also Awad et al.^11^), none of the three mutations in the ICS affected binding to PG in binding assays (Figure 3C). This suggests that the loss of PG immunoreactivity from the intercalated disk, as seen in our ARVC patients (Figure 2), is a result of a more complex mechanism than just a simple change in binding affinity caused by the missense mutation.

In particular, posttranslational modifications may be important in mediating the binding of DSG2 to PKP2. We observed striking differences in the binding properties between bacterially expressed DSG2 ICS protein and its counterpart expressed in mammalian COS-1 cells. The former bound both PG and PKP2 from rat heart lysates, whereas the latter did not bind to PKP2, even though PKP2 is abundantly expressed in this cell line. Posttranslational modifications of the DSG2 ICS region may occur in mammalian cells, which could change its affinity to PKP2. In agreement with this hypothesis, bacterially expressed DSG2 ICS was able to bind PKP2 from COS-1 cells, whereas COS-1 cell-derived DSG2 ICS did not bind to PKP2 from rat heart extracts (Online Supplement Figure 2). In addition, changes in the ICS of DSG2 may affect the structure of the adjacent regions of the cytoplasmic domain, thereby modulating their binding properties to desmosomal components.^24^

Our finding that the DSG2 C813R ICS protein fragment shows abnormal migration behavior in gel electrophoresis further supports a role for unknown functions beyond linking DSG2 to PG and PKP2 (Figure 3E). This is only evident when the cysteine residue is mutated to arginine but not if it is changed to alanine. This suggests that the observed change of electrophoretic properties is caused by the presence of the arginine at this position rather than by the lack of cysteine (“gain of function”). Currently, the nature of this phenomenon is unclear. Phosphatase treatment did not alter electrophoretic mobility of the proteins (Online Supplement Figure 3), hence an involvement of protein phosphorylation is unlikely. Alternatively, the GCCS motif is a consensus site for protein palmitoylation,^25^ and this posttranslational modification may modulate the functions of DSG2.^26^ Finally, a direct modification of this arginine at position 813 (e.g. by methylation^27^) may occur.

Future investigations are needed to identify these additional functions of the DSG2 molecule and thereby provide novel insights into ARVC. This study suggests that the loss of PG immunoreactivity at the intercalated disk is the result of complex molecular alterations in the myocardium, which is consistent with the fact that the immunoreactive signal for PG is reduced in the vast majority of ARVC cases, even in those where no mutation in a known ARVC disease gene could be identified.^4^ This observation renders the redistribution of PG part of a final common pathway in ARVC pathogenesis,^28^ not merely stemming from disruptions in binding affinities within the desmosomal plaque.

The elucidation of these pathologic mechanisms is the next step in defining pathogenesis and developing novel therapeutic targets for patients with ARVC.

## Figures and Tables

**Table 1:** Clinical data of arrhythmogenic right ventricular cardiomyopathy patients with novel DSG2 mutations according to the revised task force criteria^13^

Patient	Sex	Age (years)	Events	Genotype	FH/Gen	Depol	Repol	Fun/Str	Arr	Tissue	TFC
A.1	M	46	palpitations	DSG2 G812S	0	0	0	1M	1m	1M	2M + 1m
A.2	M	58	none	WT	1M	0	0	0	n/a	n/a	1M
A.3	F	43	palpitations	DSG2 G812S	1M	0	0	1M	1m	1M	3M + 1m
A.4	F	17		WT	1M	0	0	0	n/a	n/a	1M
A.5	M	15	none	DSG2 G812S	1M	0	0	1M	n/a	n/a	2M
A.6	F	35		WT	0	0	0	0	n/a	n/a	0
A.7	F	13	none	DSG2 G812S	1M	0	0	1M	n/a	n/a	2M
A.8	M	15		WT	1M	0	0	1m	n/a	n/a	1M + 1m
A.9	M	30		WT	0	0	0	0	n/a	n/a	0
A.10	M	56		WT	1M	0	0	0	n/a	n/a	1M

B.1	M	76	presyncope	DSG2 C813R	0	1M	n/a	1m	1M	n/a	2M + 1m
B.2	M	57	none	WT	1M	0	n/a	0	0	n/a	1M

Patients are numbered according to the pedigrees shown in Figure 1. Any known symptoms are listed. The genotypes for the desmoglein-2 (DSG2) mutations are given.

The clinical evaluation is divided into following categories: FH/Gen = family history/genetics; Depol = depolarization/conduction abnormalities; Repol = repolarization abnormalities; Fun/Str = global and/or regional dysfunction and structural alterations; Arr = arrhythmias; Tissue = tissue characterization; TFC = summary of the number of major and minor diagnostic criteria for each patient (task force criteria). For each of the categories, whether the patient fulfils major (M), minor (m), or no (0) diagnostic criteria is given.

N/A = data not available; WT = wild-type.
