# Supplementary material for: Novel missense mutations in exon 15 of desmoglein-2: Role of the intracellular cadherin segment in arrhythmogenic right ventricular cardiomyopathy?
Source: Heart Rhythm. 2010 Oct;7(10):1446–53. doi: 10.1016/j.hrthm.2010.08.007 (PMC2994644; doi:10.1016/j.hrthm.2010.08.007)
Supplement: Supplementary material [file mmc1.pdf]

# Novel missense mutations in exon 15 of desmoglein 2 – a role of the intracellular cadherin segment in arrhythmogenic right ventricular cardiomyopathy?

Katja Gehmlich, Angeliki Asimaki, Thomas Cahill, Elisabeth Ehler, Petros Syrris, Elisabetta Zachara, Federica Re, Andrea Avella, Lorenzo Monserrat, Jeffrey E. Saffitz, William J. McKenna

## Supplementary Methods

### Clinical evaluation of the families

Clinical evaluation included history, physical examination, 12-lead electrocardiogram (ECG), 2-dimensional echocardiography with accurate right ventricular (RV) assessment, and ambulatory ECG monitoring.

### Genetic screening

Genomic DNA was extracted from whole blood using QIAamp DNA Blood mini kits (Qiagen). For screening of DSP, PG, PKP2, DSG2, DSC2 and Cx43, primer pairs for all exons were used from flanking intronic sequences[1]. Polymerase chain reaction (PCR) amplification was carried out using standard protocols (AmpliTaq Gold, Applied Biosystems), and fragments with high GC content were amplified with the GC RICH PCR system (Roche). All primer sequences and PCR conditions are available on request. PCR products were subjected to direct sequencing in both directions on an Applied Biosystems 3130 Genetic Analyzer using BigDye Terminator chemistry (v3.1) and analyzed by Seqscape

v2.5 software (Applied Biosystems). A total of 400 unrelated healthy, ethnically matched volunteers served as controls.

**Histological and immuno-histochemical analysis of endomyocardial biopsies**

Right ventricular biopsy samples obtained from the proband (A.1) and his sister (A.3) during a right heart catheterization were kindly provided by Dr Giulia D'Amati (Department of Experimental Medicine, La Sapienza University, Rome, Italy). Myocardial specimens obtained at autopsy from three age-matched individuals with no clinical history or pathological evidence of heart disease were subjected to the same staining protocols and served as controls.

Histological analysis (Masson's trichrome stain) was by standard methods. For immuno-histochemical analysis, slide-mounted sections were deparaffinised, placed in citrate buffer (10 mmol/l, pH 6.0), and heated in a microwave oven until boiling for 10 min to enhance specific immunostaining. After being cooled to room temperature, the tissue sections were simultaneously permeabilised and blocked by incubating them in PBS containing 0.1% Triton X-100 and 3% normal goat serum. The sections were then incubated with the primary antibody overnight at 4°C, brought to room temperature, washed three times in phosphate buffered saline (PBS), and incubated with indocarbocyanine-conjugated goat anti-mouse or anti-rabbit IgGs (Jackson Immunoresearch) for 2 h at 25° C. Primary antibodies included polyclonal rabbit anti-connexin43 (Cx43) (Sigma), monoclonal mouse anti-PG (Sigma), polyclonal rabbit anti-DSP (SeroTec), monoclonal mouse anti-N-cadherin (Sigma), and monoclonal mouse anti-PKP2 (BioDesign) antibodies. Immunostained preparations were analyzed by laser-scanning confocal microscopy (Sarastro Model 2000, Molecular Dynamics) as previously described[2].

**Functional studies**

For localization and expression studies, the sequence of human full length DSG2 was cloned into pEGFP-N1 (Clontech) using XhoI and BamHI restriction sites. The mutations were introduced into the constructs with the QuikChange mutagenesis kit (Stratagene) according to the manufacturer's instructions. Following primer pairs were used:

5'- ctgaatcgctgaatgcttctatttgttgttgcagttttattgaagg – 3' and

5'- ccttcaataaaactgcaacaacaaatagaagcattcagcgattcag – 3' for DSG2 G812C;

5' – ctgaatcgctgaatgcttctattagttgttgcagttttattgaagg -3' and

5'- ccttcaataaaactgcaacaactaatagaagcattcagcgattcag -3' for DSG2 G812S;

5' – cgctgaatgcttctattggtcgttgcagttttattgaagga – 3' and

5'- tccttcaataaaactgcaacgaccaatagaagcattcagcg -3' for DSG2 C813R;

5'- cgctgaatgcttctattggtgcttgcagttttattgaagga – 3' and

5'- tccttcaataaaactgcaagcaccaatagaagcattcagcg – 3' for DSG2 C813A.

Primary cultures of neonatal rat cardiomyocytes (NRC) were established using the Neonatal Cardiomyocyte Isolation System from Worthington (Lakewood, NJ, USA). The cells were plated and maintained as described previously[3] and transfected on day 1 after plating using 4 µg of DNA and JetPrime transfection reagent (Autogen Bioclear). 48 h after transfection, cells were fixed with 4 % paraformaldehyde in PBS for 10 min at room temperature, and subsequently permeabilised with 0.2 % Triton X-100 in PBS for 5 min. Cells were blocked with 10 % normal goat serum (Sigma) in PBS prior to incubation with the first antibody (poly-clonal rabbit against beta-catenin, Sigma) diluted in 1 % bovine serum albumin in PBS, for 1 h at room temperature. After washing with 0.1 % Tween-20 in PBS, cells were incubated with Goat-anti-Rabbit-Antibody conjugated to Cy5 (Jackson ImmunoResearch). 4',6-diamidino-2-phenylindole (DAPI, Sigma) was used to visualise nuclei. After washing, cells were mounted in Tris-buffered glycerol with n-propyl gallate

(Sigma) as anti-fading agent. Specimens were analyzed and documented on a confocal microscope Leica SPE (Leica), using a 63x/1.30 oil immersion lens.

For binding assays, wild-type and mutant DSG2 ICS sequences (amino acids 635-842 of human DSG2) were cloned into pGEX6P1 (GE Healthcare) using BamHI and NotI restriction sites, transformed into BL21 Codon Plus strain (Stratagene), expressed as described[3] and purified with glutathione-sepharose (GE Healthcare) according to the manufacturer's instructions.

Protein-loaded beads were incubated with pre-cleared adult rat heart lysate (350 µg total protein) in Glutathion-S-Transferase (GST)-pulldown buffer (0.5 % NP-40, 20 mM Tris/HCl pH 7.5, 120 mM sodium chloride, 1 mM dithiothreitol, 1 mM sodium ortho-vanadate and protease inhibitors) for 1h on ice. After washing three times with GST-pulldown buffer, the bound proteins were eluted with two-fold SDS-sample buffer. GST-fusion proteins were analyzed by SDS-PAGE and visualised with InstantBlue (Generon), binding of PG and PKP2 was detected by Western blotting as described[3] using the following antibodies: anti-PG (clone 15/γ-Catenin BD Biosciences), anti-PKP2 (multi-epitope cocktail, Progen) and appropriate secondary antibodies conjugated to horse radish peroxidase (GE Healthcare). Where indicated, the chemiluminescent signals (SuperSignal West Pico or Dura Chemiluminescent Substrate, Thermo Scientific) were recorded with the Molecular Imager Chemidoc XRS system using Quantity One software (both Biorad).

The same DSG2 ICS constructs were also cloned into the mammalian expression vector pEBG[4] (a kind gift of Dr. Alan Whitmarsh, University of Manchester, UK) as above for expression as GST-fusion proteins in COS-1 cells, which were cultured and transfected with Escort IV (Sigma) as described[5]. After 48 hours, the cells were harvested, lysed in GST-pulldown buffer for 20 min on ice, and extracts clarified by centrifugation at 50,000 g at 4°C for 15 minutes. GST-pulldown assays were performed by addition of glutathione-sepharose

for 1 h on ice and washed, eluted and analyzed as above; detection of GST fusion proteins was performed by Western blotting (polyclonal goat anti-GST antibody, GE Healthcare).

For phosphatase treatment, the cell lysates were incubated with 10 U FastAP™ Thermosensitive Alkaline Phosphatase (Fermentas) at 37°C for 60 minutes according to the manufacturer's instructions.

## Supplementary Figures

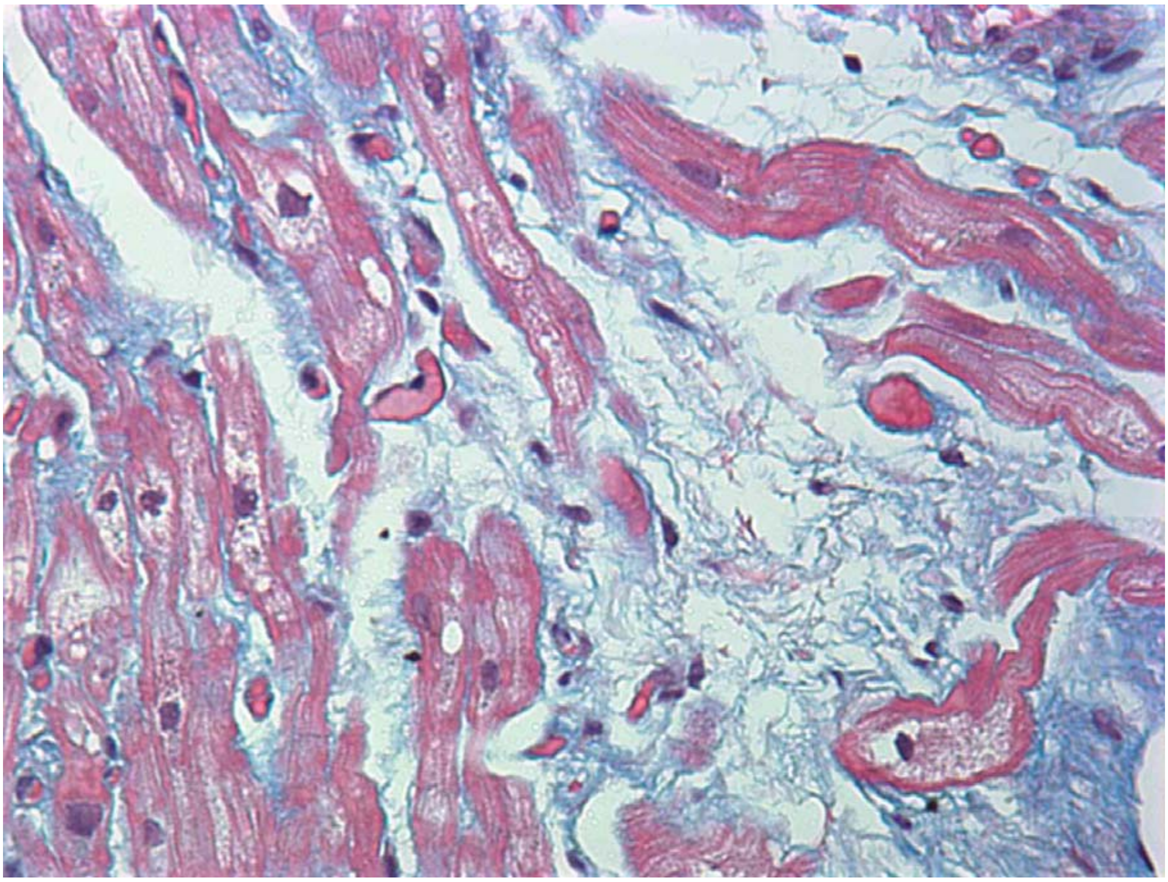

**Figure S1: Histological changes in the presence of the DSG2 G812S mutation.**

Interstitial and replacement fibrosis were observed in an endomyocardial biopsy sample obtained from the index patient (A.1) (Masson's trichrome; x40). Despite clear evidence of myocyte degeneration, no significant inflammatory or fatty infiltrates were observed. The absence of adipocytes might be due to the young age of the patient or the patchy nature of the disease. The electric isolation of surviving myocytes by surrounding scar tissue may explain the arrhythmias observed (35,000 ventricular ectopic beats on a 24 hour ECG monitor): Such substrate abnormalities are thought to facilitate re-entrant excitation in the ventricle, contributing to arrhythmia[6].

A similar histological finding for this patient has already been shown in[7].

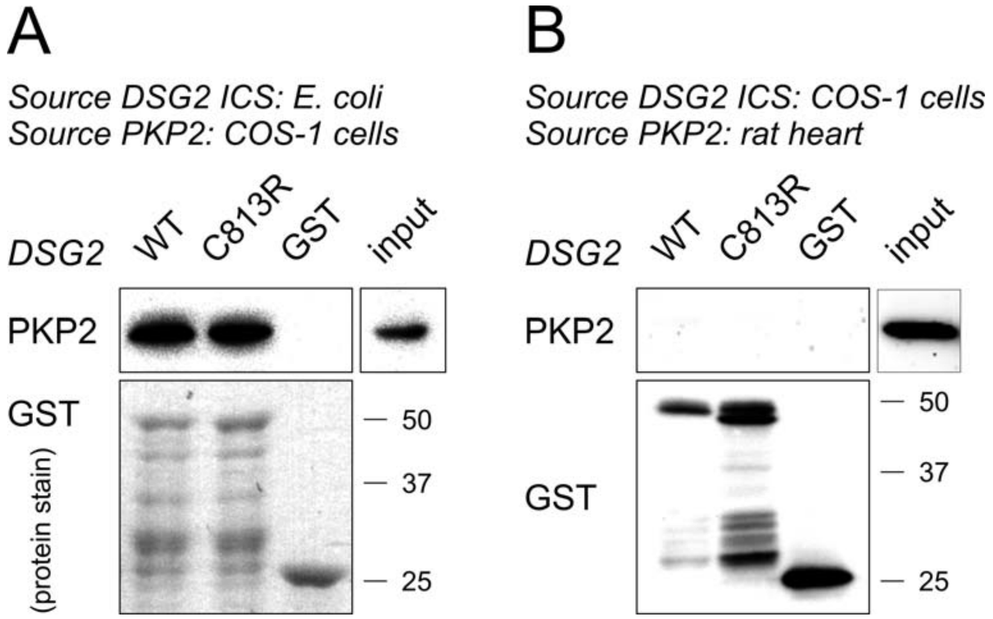


**Figure S2: The binding characteristics of DSG2 ICS protein to PKP2 depend on the expression system for the recombinant GST fusion protein.**

To address the question, why binding of DSG2 ICS (expressed in COS-1 cells) to endogenous PKP2 was not observed (Figure 3D), following GST-pulldown experiments were performed:

A – Bacterially expressed DSG2 ICS WT and C813R bound to PKP2 from COS-1 cell lysates. No binding to GST alone is shown as a negative control. Therefore, competition of PKP2 with a high affinity ligand (endogenous to COS-1 cells) for DSG2 ICS appears to be unlikely.

B – No binding of DSG2 ICS WT and C813R expressed in COS-1 cells to PKP2 from rat heart lysates was observed. In conclusion, the DSG2 ICS protein expressed in COS-1 cells is impaired in binding PKP2, independent of the source of its binding partner. Post-translational modifications occurring in mammalian cells, but not in bacteria, may regulate the binding to PKP2.

Detection of Western blot signals was performed with the Molecular Imager Chemidoc XRS system.

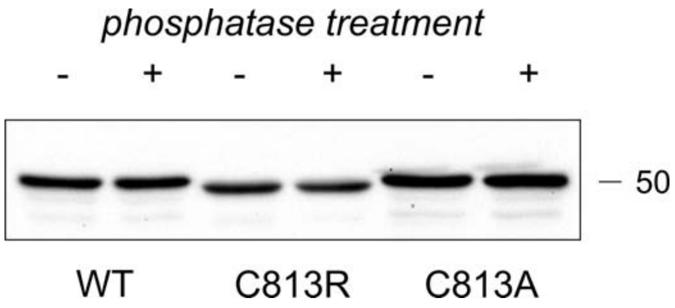


**Figure S3: Phosphatase treatment does not alter the electrophoretic mobility of DSG2 ICS proteins.**

DSG2 ICS WT, C813R and C813A were expressed as GST fusion proteins in COS-1 cells and lysates treated with alkaline phosphatase before Western blotting. This treatment did not affect the electrophoretic mobility of the proteins, suggesting that the differences observed for C813R are not caused by protein phosphorylation.

The detection of the signals was performed with Molecular Imager Chemidoc XRS system, the position of the 50 kD marker band is indicated.

## Supplementary References


1 Sen-Chowdhry S, Syrris P, McKenna WJ: Role of genetic analysis in the management of patients with arrhythmogenic right ventricular dysplasia/cardiomyopathy. J Am Coll Cardiol 2007; 50:1813-21.

2 Saffitz JE, Schuessler RB, Yamada KA: Mechanisms of remodeling of gap junction distributions and the development of anatomic substrates of arrhythmias. Cardiovasc Res 1999; 42:309-17.

3 Gehmlich K, Pinotsis N, Hayess K et al.: Paxillin and ponsin interact in nascent costameres of muscle cells. J Mol Biol 2007; 369:665-82.

4 Mayer BJ, Hirai H, Sakai R: Evidence that SH2 domains promote processive phosphorylation by protein-tyrosine kinases. Curr Biol 1995; 5:296-305.

5 Lange S, Himmel M, Auerbach D et al.: Dimerisation of myomesin: implications for the structure of the sarcomeric M-band. J Mol Biol 2005; 345:289-98.

6 Arruda M, Armaganijan L, Fahmy T, Di BL, Patel D, Natale A: Catheter ablation of ventricular tachycardia in arrhythmogenic right ventricular dysplasia. J Interv Card Electrophysiol 2009; 25:129-33.

7 Avella A, d'Amati G, Pappalardo A et al.: Diagnostic value of endomyocardial biopsy guided by electroanatomic voltage mapping in arrhythmogenic right ventricular cardiomyopathy/dysplasia. J Cardiovasc Electrophysiol 2008; 19:1127-34.
